# Differential Respiratory Responses to Incremental Positive End-Expiratory Pressure Among Healthy Adults, Cigarette Smokers, Electronic Cigarette Users, and Individuals with Asthma

**DOI:** 10.3390/diseases14070248

**Published:** 2026-07-10

**Authors:** Patchareeya Amput, Sirintip Kumfu, Sirima Wongphon

**Affiliations:** 1 Department of Physical Therapy, School of Allied Health Sciences, University of Phayao, Phayao 56000, Thailand; sirintip.ku@up.ac.th; 2 Unit of Excellence of Human Performance and Rehabilitations, University of Phayao, Phayao 56000, Thailand; 3 Department of Traditional Chinese Medicine, School of Public Health, University of Phayao, Phayao 56000, Thailand; sirima.wo@up.ac.th

**Keywords:** positive end-expiratory pressure, respiratory mechanics, cigarette smoking, electronic cigarette, asthma, thoracoabdominal motion, lung aeration

## Abstract

Background: Positive end-expiratory pressure (PEEP) influences respiratory mechanics, ventilation distribution, and lung aeration. However, comparative responses to incremental PEEP among healthy individuals, cigarette smokers, electronic cigarette users, and individuals with asthma remain poorly understood. This study aimed to characterize differential respiratory responses to incremental PEEP across these populations. Methods: A secondary analysis was conducted using an open-access respiratory physiology dataset obtained from PhysioNet. Eighty adults were included and categorized into four groups (n = 20 per group): healthy controls, cigarette smokers, electronic cigarette users, and individuals with asthma. Airway pressure, respiratory flow, tidal volume, chest circumference, abdominal circumference, and global aeration were evaluated across incremental PEEP levels ranging from 4 to 12 cmH_2_O. Results: Increasing PEEP significantly affected all respiratory and aeration outcomes (all *p* < 0.001). Significant Group × PEEP interactions were observed for airway pressure (*p* < 0.001), respiratory flow (*p* = 0.009), chest circumference (*p* = 0.002), and abdominal circumference (*p* = 0.005), indicating differential physiological responses among groups. In contrast, tidal volume (*p* = 0.067) and global aeration (*p* = 0.170) demonstrated similar response patterns across groups despite significant overall PEEP effects. Conclusions: Incremental PEEP significantly influenced respiratory mechanics, thoracoabdominal motion, and lung aeration. Respiratory responses differed among healthy controls, cigarette smokers, electronic cigarette users, and individuals with asthma, suggesting group-specific adaptations to increasing PEEP.

## 1. Introduction

Positive end-expiratory pressure (PEEP) is a critical determinant of alveolar recruitment, gas exchange, and ventilation distribution [1,2]. When applied incrementally, PEEP can reveal differences in respiratory pressure, airflow, tidal volume, and regional aeration responses that may remain undetectable under resting conditions [3,4]. In healthy adults, incremental PEEP promotes alveolar recruitment and improves ventilation distribution before excessive pressure levels contribute to overdistension [5,6]. Consequently, physiological responses to incremental PEEP provide a useful framework for evaluating respiratory adaptation across different populations. Furthermore, incremental PEEP may function as a physiological challenge that facilitates detection of subtle respiratory abnormalities.

Cigarette smoking, affecting approximately 1.3 billion individuals globally [7], leads to small airway remodeling, loss of elastic recoil, and parenchymal destruction. These changes manifest as increased airway resistance and prolonged time constants, predisposing individuals to dynamic hyperinflation [8,9,10,11]. Importantly, such mechanical abnormalities may precede overt airflow obstruction and may coexist with mild spirometric abnormalities, including preserved ratio impaired spirometry (PRISm), before the development of chronic obstructive pulmonary disease [12,13]. While cigarette smoking has well-established effects on respiratory mechanics, the impact of electronic cigarette use remains less clearly defined. Electronic cigarettes are now used by over 82 million individuals worldwide [14]. However, emerging evidence demonstrates that electronic cigarette aerosols induce oxidative stress, epithelial injury, impaired mucociliary clearance, and surfactant dysfunction [15,16,17,18]. These alterations may adversely affect respiratory mechanics and modify the physiological response to incremental PEEP.

Asthma, affecting approximately 363 million individuals globally and responsible for 442,000 deaths annually [19], further complicates respiratory mechanics through bronchial hyperresponsiveness, airway smooth muscle hypertrophy, and marked regional heterogeneity. These features contribute to exaggerated pressure-volume nonlinearity, airflow limitation, and paradoxical ventilation distribution under increased mechanical loading [20,21].

Despite strong mechanistic plausibility, comparative data on PEEP-mechanics relationships across these populations remain limited, particularly in non-intubated or subclinical groups. Previous studies have primarily focused on single disease categories in critically ill, invasively ventilated patients [22,23]. Advances in open-access respiratory signal databases, such as PhysioNet, now enable high-resolution breath-by-breath analysis of respiratory pressure, airflow, tidal volume, chest wall motion, abdominal motion, and regional lung aeration responses during controlled physiological interventions [24]. These datasets provide an opportunity to investigate respiratory adaptations to incremental PEEP with substantially greater temporal resolution than conventional pulmonary function testing.

The present study aimed to systematically characterize differential respiratory responses in airway pressure, respiratory flow, tidal volume, chest excursion, abdominal excursion, and global aeration during incremental PEEP across four populations: healthy adults, cigarette smokers, electronic cigarette users, and individuals with asthma. We hypothesized that cigarette smokers and individuals with asthma would demonstrate attenuated compliance responses and greater resistance to PEEP-induced recruitment compared with healthy controls, whereas electronic cigarette users would exhibit intermediate patterns reflecting early subclinical airway dysfunction.

## 2. Materials and Methods

### 2.1. Study Design

This study employed a secondary analysis design using publicly available respiratory physiology data obtained from PhysioNet [24]. The original dataset was prospectively collected under a standardized incremental positive end-expiratory pressure (PEEP) protocol. Secondary analysis was conducted to evaluate differential respiratory and aeration responses across four participant groups: healthy adults, cigarette smokers, electronic cigarette users, and individuals with asthma. Comparative analyses across PEEP levels were performed to identify between-group differences in adaptation to incremental PEEP.

### 2.2. Participants

The study utilized data from an open-access respiratory physiology dataset available through PhysioNet [24]. The dataset included 80 adult participants distributed equally across four groups (n = 20 per group): healthy controls, cigarette smokers, electronic cigarette users, and individuals with asthma. Healthy participants had no history of respiratory disease and demonstrated normal pulmonary function. Participants in the cigarette smoker group were classified according to the original PhysioNet dataset as current cigarette smokers. Detailed smoking exposure characteristics, including pack-years and smoking duration, were not available in the publicly accessible dataset. Electronic cigarette users were classified according to the original PhysioNet dataset as participants who regularly used electronic cigarettes. Detailed information regarding previous conventional cigarette smoking, dual use, smoking cessation status, duration of electronic cigarette use, and nicotine exposure was not available in the publicly accessible dataset. Participants in the asthma group were classified based on a physician-confirmed diagnosis of asthma as reported in the original dataset; information regarding smoking history within this group was unavailable. Due to the secondary nature of the dataset, detailed information on disease severity, duration of exposure, and comorbidities was limited and could not be fully controlled in the analysis.

### 2.3. Procedure

The primary outcome variables included airway pressure (cmH_2_O), respiratory flow (L/s), tidal volume (L), chest excursion (mm), abdominal excursion (mm), and global aeration derived from electrical impedance tomography. Measurements were evaluated across incremental PEEP levels ranging from 4 to 12 cmH_2_O.

#### 2.3.1. Data Acquisition

Respiratory waveform data were obtained from a publicly available respiratory physiology dataset hosted on PhysioNet [24]. Airway pressure, respiratory flow, tidal volume, chest excursion, abdominal excursion, and global aeration were recorded by the original investigators using a standardized respiratory monitoring system during a controlled incremental PEEP protocol. Incremental PEEP was applied according to the original experimental protocol, with measurements acquired at successive PEEP levels ranging from 4 to 12 cmH_2_O after stabilization at each level. The present study analyzed the processed physiological signals provided in the original dataset; detailed technical specifications of the measurement devices and the PEEP delivery system are described in the original dataset publication.

#### 2.3.2. Signal Processing and Respiratory Response Analysis

Respiratory waveform signals were processed using the standardized pipeline provided with the PhysioNet dataset. Airway pressure, respiratory flow, tidal volume, chest displacement, abdominal displacement, and global aeration signals were synchronized and analyzed across incremental PEEP levels. Individual respiratory cycles were identified using inspiratory indices provided in the processed dataset, allowing breath-by-breath characterization of respiratory responses.

#### 2.3.3. Ethical Considerations

This study utilized a publicly available, de-identified respiratory physiology dataset obtained from PhysioNet. All data were anonymized prior to public release, and no personally identifiable information was accessible to the investigators. The original study received ethical approval from the Human Research Ethics Committee at the University of Canterbury (Reference No. HREC 2023/04/LR-PS), with amendments approved on 24 March 2023 [23]. Because the present study involved a secondary analysis of a publicly available, fully de-identified dataset and did not involve direct participant recruitment or contact, additional ethical approval and participant informed consent for the present analysis were not required. The study adhered to the principles of the Declaration of Helsinki and complied with PhysioNet data-use policies.

### 2.4. Statistical Analysis

Given the secondary nature of this analysis, a formal a priori power calculation was not performed, as the sample size was determined by the original dataset (n = 20 per group). Nevertheless, this sample size is consistent with prior respiratory physiology studies employing repeated-measures designs and high-resolution respiratory signal analysis, in which within-subject measurements across multiple PEEP levels substantially increase statistical power relative to between-subject comparisons alone. The use of linear mixed-effects modeling further optimizes the utilization of all available observations, thereby enhancing the precision of parameter estimates. These considerations suggest that the available sample may be sufficient to detect moderate repeated-measures effects; however, the absence of an a priori power calculation should be considered when interpreting nonsignificant findings. Descriptive statistics were calculated for all study variables. Respiratory parameters, including airway pressure, respiratory flow, tidal volume, chest excursion, abdominal excursion, and global aeration, were summarized as mean ± standard deviation (SD), as appropriate. Separate linear mixed-effects models were constructed for each respiratory outcome variable. Group (healthy, smoker, electronic cigarette user, and asthma), PEEP level, and the group-by-PEEP interaction were specified as fixed effects, while participant identity was included as a random intercept to account for repeated measurements within subjects. Estimated marginal means with Bonferroni adjustment were used for pairwise comparisons among participant groups when appropriate. Pairwise comparisons were not performed across individual PEEP levels because PEEP was modeled as a continuous variable in the linear mixed-effects models. All statistical analyses were performed using IBM SPSS Statistics, version 26.0, with the significance level set at *p* < 0.05.

## 3. Results

Baseline demographic characteristics did not differ significantly among the four groups (Table 1). Age, sex distribution, height, weight, and body mass index were comparable across healthy controls, cigarette smokers, electronic cigarette users, and individuals with asthma (all *p* > 0.05).

Incremental PEEP exerted a significant main effect on all respiratory and aeration outcomes (all *p* < 0.001; Table 2), indicating that increasing PEEP elicited measurable physiological responses across participants. Significant Group × PEEP interactions were identified for airway pressure (F = 15.638, *p* < 0.001), respiratory flow (F = 3.880, *p* = 0.009), chest excursion (F = 4.819, *p* = 0.002), and abdominal excursion (F = 4.257, *p* = 0.005), demonstrating that the pattern of change across PEEP levels differed among groups for these outcomes. In contrast, the Group × PEEP interactions for tidal volume (F = 2.396, *p* = 0.067) and global aeration (F = 1.679, *p* = 0.170) were not statistically significant, suggesting broadly similar PEEP-related trends across groups for these variables.

**Figure 1:**
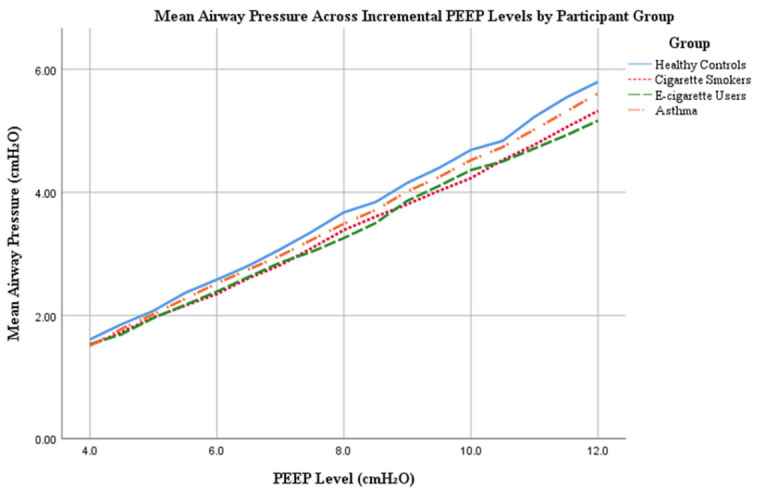
Mean airway pressure across incremental positive end-expiratory pressure (PEEP) levels in healthy controls, cigarette smokers, electronic cigarette users, and individuals with asthma. Values represent group means at each PEEP level. Airway pressure increased progressively with increasing PEEP in all groups, with distinct response patterns observed among participant groups.

Figure 1 illustrates mean airway pressure responses across incremental PEEP levels in healthy controls, cigarette smokers, electronic cigarette users, and individuals with asthma. Mean airway pressure increased progressively with increasing PEEP in all groups, demonstrating a strong effect of PEEP on airway pressure regulation (F(1, 1035.14) = 23,735.44, *p* < 0.001). A significant Group × PEEP interaction was observed (F(3, 1035.14) = 15.638, *p* < 0.001), indicating that the rate of increase in airway pressure differed among groups. Across the entire PEEP range, healthy controls consistently exhibited the highest mean airway pressure values, whereas electronic cigarette users showed the lowest values. Participants with asthma and cigarette smokers demonstrated intermediate responses. Although airway pressure increased in all groups as PEEP was progressively elevated, the separation between groups became more apparent at higher PEEP levels, particularly above 8 cmH_2_O, suggesting differential pressure adaptation to increasing ventilatory load.

**Figure 2:**
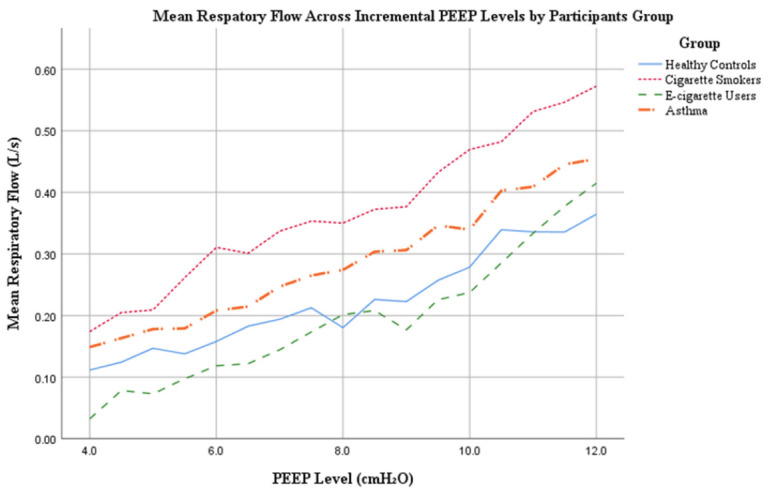
Mean respiratory flow across incremental positive end-expiratory pressure (PEEP) levels in healthy controls, cigarette smokers, electronic cigarette users, and individuals with asthma. Values represent group means at each PEEP level. Respiratory flow increased with increasing PEEP, with differences in flow adaptation observed among the four study groups.

Figure 2 presents mean respiratory flow responses across incremental PEEP levels in healthy controls, cigarette smokers, electronic cigarette users, and individuals with asthma. Respiratory flow increased progressively with increasing PEEP in all groups, confirming a significant main effect of PEEP (F(1, 999.77) = 518.059, *p* < 0.001). A significant Group × PEEP interaction was observed (F(3, 999.77) = 3.880, *p* = 0.009), indicating that the magnitude of flow adaptation to increasing PEEP differed among groups. Across the entire PEEP range, cigarette smokers exhibited the highest respiratory flow values, followed by individuals with asthma. Healthy controls and electronic cigarette users generally demonstrated lower flow responses, although respiratory flow increased in all groups as PEEP increased. The divergence between groups became more pronounced at higher PEEP levels, particularly above 9 cmH_2_O, suggesting differential ventilatory responses to increasing airway pressure.

**Figure 3:**
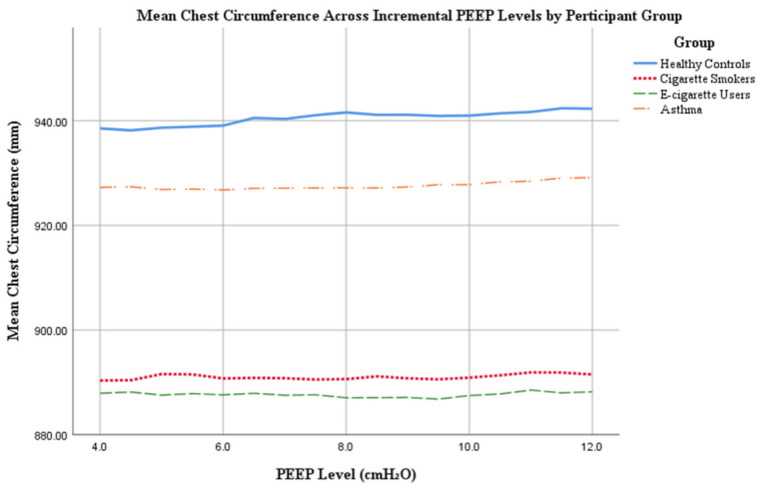
Mean chest circumference across incremental positive end-expiratory pressure (PEEP) levels in healthy controls, cigarette smokers, electronic cigarette users, and individuals with asthma. Values represent group means at each PEEP level. Chest circumference increased modestly with increasing PEEP, with distinct thoracic expansion patterns observed among participant groups.

Figure 3 illustrates mean chest circumference across incremental PEEP levels in healthy controls, cigarette smokers, electronic cigarette users, and individuals with asthma. Chest circumference was significantly influenced by increasing PEEP (F(1, 780.74) = 39.313, *p* < 0.001). A significant Group × PEEP interaction was also identified (F(3, 780.74) = 4.819, *p* = 0.002), indicating that thoracic expansion responses to PEEP differed among groups. Healthy controls consistently demonstrated the largest chest circumference values across all PEEP levels, whereas electronic cigarette users exhibited the smallest values. Participants with asthma showed intermediate chest circumference values, while cigarette smokers demonstrated slightly greater values than electronic cigarette users. Although chest circumference increased only modestly with increasing PEEP, the relative differences among groups remained evident throughout the protocol, reflecting distinct thoracic expansion patterns in response to increasing airway pressure.

**Figure 4:**
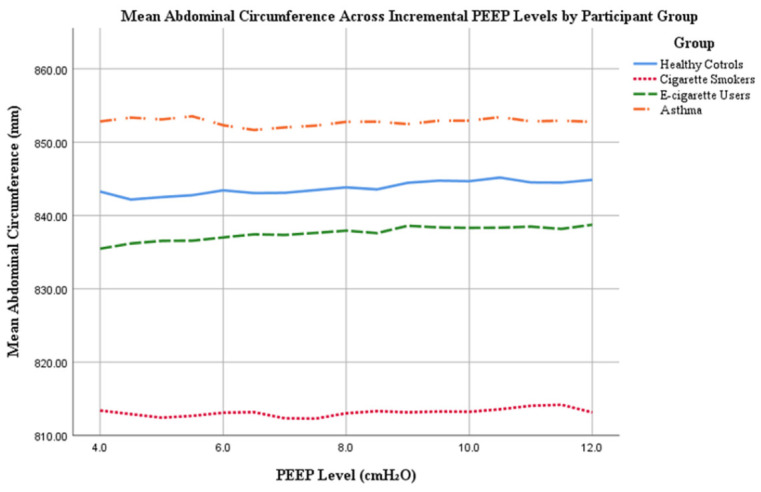
Mean abdominal circumference across incremental positive end-expiratory pressure (PEEP) levels in healthy controls, cigarette smokers, electronic cigarette users, and individuals with asthma. Values represent group means at each PEEP level. Abdominal circumference increased modestly with increasing PEEP, with group-specific abdominal motion responses observed throughout the protocol.

Figure 4 depicts mean abdominal circumference responses across incremental PEEP levels in healthy controls, cigarette smokers, electronic cigarette users, and individuals with asthma. Abdominal circumference increased significantly with increasing PEEP (F(1, 930.15) = 50.197, *p* < 0.001). A significant Group × PEEP interaction was observed (F(3, 930.15) = 4.257, *p* = 0.005), indicating that abdominal expansion patterns in response to increasing PEEP differed among groups. Across all PEEP levels, individuals with asthma demonstrated the largest abdominal circumference values, whereas cigarette smokers consistently exhibited the lowest values. Healthy controls and electronic cigarette users showed intermediate values throughout the protocol. Although the overall increase in abdominal circumference with increasing PEEP was relatively modest, distinct group-specific response patterns were maintained across the entire PEEP range, supporting differential abdominal motion adaptations to progressive airway pressure loading.

Increasing PEEP significantly altered respiratory mechanics and thoracoabdominal motion, and the magnitude of these responses differed among healthy controls, cigarette smokers, electronic cigarette users, and individuals with asthma.

## 4. Discussion

The present study investigated the effects of incremental positive end-expiratory pressure (PEEP) on respiratory mechanics, thoracoabdominal motion, and lung aeration in healthy controls, cigarette smokers, electronic cigarette users, and individuals with asthma. The principal findings were that (1) increasing PEEP significantly affected all measured respiratory and aeration outcomes, (2) airway pressure, respiratory flow, chest excursion, and abdominal excursion exhibited significant Group × PEEP interactions, and (3) tidal volume and global aeration demonstrated similar response patterns across groups despite significant overall effects of PEEP.

As expected, airway pressure increased progressively with increasing PEEP in all groups. This finding is consistent with the physiological role of PEEP in maintaining positive airway pressure throughout the respiratory cycle and increasing end-expiratory lung volume [2]. However, the significant Group × PEEP interaction suggests that the magnitude of pressure adaptation differed among healthy controls, cigarette smokers, electronic cigarette users, and individuals with asthma. These findings indicate that respiratory responses to externally applied positive pressure vary according to participant group and may reflect differences in respiratory system behavior associated with smoking exposure and asthma [25,26,27].

Respiratory flow also increased significantly with increasing PEEP and demonstrated a significant interaction between group and PEEP level. Interestingly, cigarette smokers exhibited the highest respiratory flow values across most PEEP levels. Although the underlying mechanisms cannot be determined from the present dataset, the observed interaction suggests that the flow response to progressive PEEP loading differed among groups. Similarly, individuals with asthma demonstrated flow responses that differed from those of healthy controls, indicating that respiratory adaptation to increasing airway pressure may not be uniform across populations with different respiratory backgrounds [9,28,29,30,31,32].

Thoracoabdominal motion measurements provided additional insight into respiratory adaptation. Healthy controls consistently demonstrated greater chest excursion than the other groups, whereas individuals with asthma exhibited the greatest abdominal excursion. These findings may suggest that respiratory muscle recruitment patterns may differ according to respiratory health status; however, respiratory muscle activity was not directly assessed in the present study [33,34]. Greater chest wall movement in healthy individuals may reflect more efficient thoracic expansion during increasing PEEP [33], whereas increased abdominal motion in asthma may suggest the possibility of greater reliance on diaphragmatic breathing and compensatory respiratory muscle activity [35,36]. Because diaphragmatic function and respiratory muscle activity were not directly measured, these interpretations remain speculative. The significant Group × PEEP interactions observed for both chest and abdominal excursion may be consistent with different breathing strategies among groups during progressive airway pressure loading; however, this interpretation should be considered hypothesis-generating rather than confirmatory.

In contrast, tidal volume did not demonstrate a statistically significant Group × PEEP interaction, despite a significant overall effect of PEEP. This finding suggests that although tidal volume increased with increasing PEEP, the overall pattern of change was relatively similar across groups. Likewise, global aeration did not exhibit a significant interaction between group and PEEP level. Taken together, these findings suggest that while pressure, flow, and thoracoabdominal motion responded differently among groups, the overall effects of PEEP on tidal volume and aeration followed comparable trajectories across the study populations [33]. However, interpretation of the aeration findings should be undertaken cautiously because aeration measurements were available only up to PEEP 8 cmH2O in the source dataset, and values beyond this level were recorded as zero.

The present findings have potential clinical implications. The observation that respiratory responses to increasing PEEP differed among healthy individuals, smokers, electronic cigarette users, and individuals with asthma suggests that respiratory system adaptations to positive-pressure loading may be influenced by underlying pulmonary status and exposure history. These differences may be relevant when considering individualized respiratory assessment or the physiological effects of positive airway pressure interventions.

### Clinical Implications

Although the present study was not designed to establish optimal PEEP settings or guide clinical respiratory management, the observed group-specific responses to incremental PEEP suggest that physiological adaptation to positive airway pressure differs according to respiratory health status. These findings may assist future investigations aimed at identifying early respiratory mechanical alterations and developing individualized respiratory assessment strategies. The intermediate physiological responses observed in electronic cigarette users may indicate subtle respiratory adaptations that are not as pronounced as those in cigarette smokers but differ from healthy individuals, highlighting the need for further longitudinal studies to determine their clinical significance. Future prospective studies incorporating comprehensive pulmonary function testing, smoking exposure assessment, and disease severity measures are required before these findings can be translated into clinical decision-making or respiratory rehabilitation protocols.

Several limitations should be acknowledged. First, the study was based on a secondary analysis of an open-access dataset, limiting control over data acquisition procedures and participant selection. Second, demographic and clinical variables available in the dataset were limited, restricting adjustment for potential confounding factors. Third, aeration measurements were incomplete at higher PEEP levels, which may have reduced sensitivity for detecting group differences in lung recruitment. Fourth, detailed smoking exposure characteristics, including pack-years, smoking duration, former smoking history, dual-use status, smoking history among participants with asthma, and the duration and frequency of electronic cigarette use, were unavailable in the source dataset. Consequently, residual confounding related to tobacco exposure cannot be excluded, and the observed findings should be interpreted with appropriate caution. However, the available data do not permit assessment of the magnitude or direction of any potential confounding effects. Future prospective studies incorporating standardized data collection, comprehensive smoking exposure assessment, pulmonary function testing, and longitudinal follow-up are warranted to validate and extend these preliminary findings and to better define their clinical relevance. Finally, the cross-sectional design precludes conclusions regarding causality or long-term physiological adaptations. In addition, the present study did not directly assess pulmonary function, airway resistance, or lung compliance; therefore, mechanistic explanations regarding these physiological characteristics should be interpreted cautiously.

## 5. Conclusions

Increasing PEEP significantly influenced respiratory mechanics, thoracoabdominal motion, and lung aeration. Furthermore, healthy controls, cigarette smokers, electronic cigarette users, and individuals with asthma exhibited distinct pressure, flow, and thoracoabdominal responses to progressive PEEP loading, highlighting differences in respiratory system adaptation among these populations.

## Figures and Tables

**Table 1:** Baseline demographic and anthropometric characteristics of the study participants.

Variables	Healthy (n = 20)	Cigarette Smokers (n = 20)	E-Cigarette Users (n = 20)	Asthma (n = 20)	*p*-Value
Age (years)	24.25 ± 4.05	24.00 ± 2.87	22.25 ± 0.91	25.35 ± 8.13	0.238
Sex, n (male/female)	10/10	10/10	10/10	10/10	1.000
Height (m)	1.74 ± 0.07	1.75 ± 0.11	1.77 ± 0.12	1.72 ± 0.11	0.507
Weight (kg)	75.09 ± 15.64	77.51 ± 23.64	76.11 ± 13.49	73.70 ± 14.42	0.914
BMI (kg/m^2^)	24.74 ± 4.35	24.95 ± 6.11	24.28 ± 3.27	25.01 ± 4.93	0.962

Denote: m = meter; kg = kilogram; kg/m^2^ = kilogram per square meter; BMI = body mass index. Values are presented as mean ± standard deviation (SD) unless otherwise indicated. Sex is presented as frequency (n). *p*-Values were derived from one-way analysis of variance (ANOVA) for continuous variables and Pearson’s chi-square test for categorical variables.

**Table 2:** Results of linear mixed-effects models examining the effects of group, PEEP level, and their interaction on respiratory and aeration parameters.

Outcome Variable	Group F (*p*)	PEEP F (*p*)	Group × PEEP F (*p*)
Airway pressure	0.332 (0.803)	23,735.442 (<0.001 ***)	15.638 (<0.001 ***)
Respiratory flow	0.332 (0.802)	518.059 (<0.001 ***)	3.880 (0.009 **)
Tidal volume	0.497 (0.685)	340.117 (<0.001 ***)	2.396 (0.067)
Chest excursion	0.493 (0.688)	39.313 (<0.001 ***)	4.819 (0.002 **)
Abdominal excursion	0.249 (0.862)	50.197 (<0.001 ***)	4.257 (0.005 **)
Global aeration	2.006 (0.118)	22.687 (<0.001 ***)	1.679 (0.170)

*** *p* < 0.001; ** *p* < 0.01. Values are F statistics with *p*-values in parentheses. Group comprised healthy controls, cigarette smokers, electronic cigarette users, and individuals with asthma.

## Data Availability

The data presented in this study are available in PhysioNet at https://doi.org/10.13026/d767-e709 (Respiratory dataset from PEEP study with expiratory occlusion, version 1.0.0). The data used in this study were derived from the publicly available PQ_rawData directory (accessed on 1 June 2026).
